# Schulische Gesundheitsförderung von Ottawa bis heute: Chancen und Herausforderungen

**DOI:** 10.1007/s00103-022-03550-x

**Published:** 2022-06-01

**Authors:** Peter Paulus

**Affiliations:** Zentrum für Angewandte Gesundheitswissenschaften, Leuphana Universität Lüneburg, Wilschenbrucher Weg 84a, 21335 Lüneburg, Deutschland

**Keywords:** Praxisfeld, Forschungsfeld, Politikfeld, Gute Gesunde Schule, Schulisches Gesundheitsmanagement, Field of practice, Field of reserarch, Field of politics, Good Healthy School, School health management

## Abstract

Ein Blick zurück auf die inzwischen 30-jährige Geschichte der schulischen Gesundheitsförderung in Deutschland lässt Entwicklungslinien erkennen, die zu ihrem Verständnis hilfreich sind und den Blick für zukünftige Chancen und Herausforderungen öffnen. In diesem Beitrag wird die schulische Gesundheitsförderung aus 3 Perspektiven betrachtet: als Praxis‑, Forschungs- und Politikfeld.

Im *Praxisfeld* ist die schulische Gesundheitsförderung konfrontiert mit der schon länger andauernden von gesundheitlichen Einschränkungen geprägten Situation von Schüler*innen, Lehrkräften und Schulleitungen. In einer Vielgestaltigkeit von Maßnahmen lassen sich 3 Realisierungsformen erkennen, die sich aus gemeinsamen Wurzeln herausgebildet haben: (a) verhaltensbasierter Ansatz, (b) gesundheitsfördernde Schule und (c) Gute Gesunde Schule. „Gesundheitskompetenz“ und „Gesundheitskompetente Schule“ stellen aktuelle Entwicklungen dar. Im *Forschungsfeld* zeigt sich, dass die Möglichkeiten der Evaluation oft nicht ausgeschöpft werden und dass Evaluation in Settingansätzen vor großen Hürden steht. In Fragen zu Strategien der Dissemination und Implementation als weitere wichtige Forschungsfelder sind deutliche Fortschritte zu verzeichnen. Im *Politikfeld* sind wichtige Meilensteine gesetzt worden mit der Präventionsgesetzgebung von 2015, dem Präventionsleitfaden des Spitzenverbandes Bund der Krankenkassen (GKV), den Empfehlungen der Kultusministerkonferenz (KMK) 2012 und mit dem Fachkonzept der Deutschen Gesetzlichen Unfallversicherung (DGUV) von 2013. Schulgesetze der Länder und die Qualitätskonzepte guter Schulen zeigen mögliche Verknüpfungen des Gesundheitsmanagements mit dem pädagogischen Qualitätsmanagement der Schulen auf.

Im Fazit wird sichtbar, dass eine Rahmung für die schulische Gesundheitsförderung noch fehlt, die theoriegeleitet Forschung und Praxis anleitet und miteinander verbindet.

## Abstract (en)

A look back at the now 30-year history of school health promotion in Germany reveals lines of development that are helpful in understanding the past and that open the view for future opportunities and challenges. In this article, school health promotion will be considered from three perspectives: as a field of practice, a field of research, and a field of policy.

In the *practice field*, health promotion in schools is confronted with a situation characterized by long-standing restrictions that have been imposed on students, teachers, and heads of schools. These measures are many and varied, but three forms of implementation that have developed from common roots can be identified: (a) behavioural approach, (b) health promoting school, and (c) good healthy school. “Health literacy” and the “health literate school” represent recent developments. In the *research field*, it is apparent that opportunities for evaluation are often underutilized, but in settings-based approaches they also face significant hurdles. However, in questions of dissemination and implementation strategies, which are further important research fields, clear progress has been made. In the *policy field*, important milestones have been set with the Preventive Care Act of 2015, the prevention guideline of the GKV (National Association of Statutory Health Insurance Funds), the recommendations of the KMK 2012 (Standing Conference of the Ministers of Education and Cultural Affairs), and with the concept of the DGUV (German Social Accident Insurance) in 2013. School legislation of the federal states and the quality concepts of good schools show how health management can be linked with the educational quality management in schools.

In conclusion, it is evident that a framework for school health promotion that would guide and link theory-based research and practice is still lacking.

## Einleitung

Nach mehr als 35 Jahren Gesundheitsförderung, beginnend mit der 1. Internationalen Konferenz der Weltgesundheitsorganisation (WHO) zur Gesundheitsförderung in Ottawa (Kanada) und der Verabschiedung der immer noch wegweisenden Ottawa-Charta, sowie nach 30 Jahren schulischer Gesundheitsförderung in Deutschland lohnt sowohl ein Blick zurück auf die Entwicklungen als auch ein Blick auf die aktuelle Situation und auf Chancen und Herausforderungen im Feld von Schule, Gesundheit und Bildung. In diesem Beitrag werden nacheinander das Praxisfeld, das Forschungsfeld und das Politikfeld der schulischen Gesundheitsförderung betrachtet. Durchgängig wird dabei der Begriff der schulischen Gesundheitsförderung verwandt. Er dient als „Klammerbegriff“, der sowohl die schulische Prävention als auch die Gesundheitserziehung und -bildung umfasst. Diese Betrachtung ist nicht die erste ihrer Art. In verschiedenen Publikationen ist die Entwicklung der schulischen Gesundheitsförderung aus dieser Perspektive begleitet und in ihren verhaltensbezogenen bzw. strukturell-systemischen Ausprägungen bzw. Organisationsentwicklungsaspekten eingeschätzt worden [1–5], aber auch aus soziologischer (s. auch den Beitrag von Bittlingmayer und Okcu in diesem Themenheft), aus biopolitischer [6] und aus Jugendhilfesicht [7, 8].

## Schulische Gesundheitsförderung als Praxisfeld

Das Praxisfeld der schulischen Gesundheitsförderung hat eine enorme Größe. Nach der aktuellen Schulstatistik^1^ gab es in Deutschland 2020/2021 etwa 10.735.347 Schüler*innen. 940.560 Lehrkräfte waren in Voll‑, Teilzeit oder stundenweise an 40.565 allgemeinbildenden und beruflichen Schulen beschäftigt.

In diesem Praxisfeld ist die schulische Gesundheitsförderung mit vielfältigen gesundheitlichen Gegebenheiten und Problemen von Schüler*innen und deren Angehörigen sowie des Schulpersonals konfrontiert. Seit einigen Jahren liegen vermehrt verlässliche Daten hierzu vor, die hier nur kurz anzusprechen sind: Psychische und psychosoziale Auffälligkeiten bei Kindern und Jugendlichen nehmen danach schon seit den 1980er-Jahren zu, ebenso sind geschlechtsspezifische Unterschiede von Auffälligkeiten immer wieder aufgezeigt worden, wie auch ihr sozialer Gradient [9]. Übergewicht und Adipositas scheinen im Jugendalter auf hohem Niveau zu stagnieren, während Alkohol- und Tabakkonsum deutlich und über die Jahre hinweg kontinuierlich abnehmen [10, 11]. Zu den neuesten gesundheitlichen Entwicklungen in der Folge der COVID-19-Pandemie liegen erste Daten vor (u. a. [12]; im Überblick [13]). Die verfügbaren Erkenntnisse zur Gesundheit der Lehrkräfte weisen schon seit Jahren auf eine psychisch angespannte Lage dieser Berufsgruppe hin. Die Mehrzahl der Studien spricht von einem hohen Risiko von Lehrkräften für psychische und psychosomatische Beanspruchungen [14]. Auch das Wohlbefinden der Schulleitungen hat sich nach vorliegenden Daten in den letzten Jahren deutlich verringert. Schulleitungen leiden ähnlich wie die Lehrkräfte vielfach unter emotional-motivationalen Erschöpfungssyndromen [15, 16].

Die schulische Gesundheitsförderung ist in den Jahren zu einem komplexen System herangewachsen. Die Vielfalt von Akteuren im Feld der schulischen Prävention und Gesundheitsförderung ist beeindruckend. Mit Initiativen, Projekten, Programmen und Ansätzen etwa zur Ernährung, Bewegung, Lebenskompetenz, Sucht, zu Erlebens- und Verhaltensproblemen entwickeln sie präventive und gesundheitsförderliche Maßnahmen und setzten diese um. Die Maßnahmen reichen von einfach strukturierten Interventionen (z. B. Unterrichtseinheiten) über ein in Schulen weitverbreitetes auf einzelne Gesundheitsthemen bezogenes projektorientiertes Vorgehen bis hin zu komplexen Settingprojekten, die die ganze Schule betreffen, auch über sie hinausreichen und in Schulnetzwerken münden. Dazu werden je nach Größe bzw. Vielschichtigkeit der Interventionen sowohl personenbezogene als auch verhältnisbezogene Strategien eingesetzt bzw. miteinander kombiniert. Dies geschieht zumeist in einem zeitlich eng begrenzten Projektrahmen und mit z. T. wenig evidenzbasierter Begründung ihrer Wirksamkeitsannahmen.

Die Maßnahmen entstammen oft unterschiedlichen Fachdisziplinen und Forschungsrichtungen und richten sich an verschiedene Zielgruppen (in der Regel an Schüler*innen, pädagogisches Personal und Eltern) mit einem vielfach nicht einheitlichen Verständnis von Gesundheit, Gesundheitsförderung und Prävention sowie mit unterschiedlichen Zielen und mit unterschiedlicher Qualität. Neben den traditionellen Kooperationspartnern der Schule aus den Bereichen der Jugendhilfe, Öffentlicher Gesundheitsdienst, Schulpsychologie, Kinder- und Jugendpsychiatrie sowie Pädiatrie drängen auch Vertreter*innen der Gesundheitsfachbereiche wie Logopädie und Ergotherapie in das Feld der schulischen Gesundheitsförderung. Seit etwa 10 Jahren gewinnt in Deutschland auch die Schulgesundheitspflege mit den Schulgesundheitsfachkräften zunehmend an Bedeutung. Überwiegend befinden sich solche Ansätze aber noch in projektförmigen Umsetzungen [17–19].

Trotz dieser hier nur skizzierten Vielgestaltigkeit der schulischen Gesundheitsförderung, trotz des vielfach zu beobachtenden unkoordinierten Vorgehens und der parallelen Strukturen, die sie mit der Prävention und Gesundheitsförderung insgesamt teilt [20], lassen sich aktuell 3 grundlegende Realisierungsformen der schulischen Gesundheitsförderung ableiten: der verhaltensbasierte Ansatz, der Ansatz „Gesundheitsfördernde Schule“ und der Ansatz „Gute gesunde Schule“ ([21]; Tab. 1), auf die im Folgenden näher eingegangen wird.

**Table 1:** 

	Verhaltensbasierter Ansatz	Gesundheitsfördernde Schule	Gute Gesunde Schule
Ausgangspunkt	Gesundheitliche Problemstellung	Gesundheitliche Problemstellung	Schulpädagogische Problemstellung
Zielgruppe	Einzelne Personengruppen (z. B. Schüler*innen)	Alle schulischen Personengruppen	Alle schulischen Personengruppen
Sichtweise von Schule	Schule als Ort, an dem die Zielgruppe erreicht werden kann	Schule als Setting, das gesundheitsförderlich gestaltet werden kann	Schule als Institution des Bildungswesens mit Bildungs- und Erziehungsauftrag
Konzept	Gesundheitsförderung in der Schule	Gesundheitsförderung durch die Schule	Bildungsförderung der Schule durch Gesundheit
Motto	Gesundheit zum Thema einzelner Zielgruppen machen	Gesundheit zum Thema der Schule machen	Mit Gesundheit gute Schule entwickeln
Strategie	Veränderung personaler Determinanten von Gesundheit	Veränderung strukturell-systemischer und personaler Determinanten von Gesundheit	Veränderung strukturell-systemischer und personaler Determinanten von Bildung durch Gesundheitsinterventionen
Outcomes	Gesundheitsbezogene(s) Wissen, Einstellungen, Verhalten der Zielgruppe(n) Gesundheitsbezogene Lebensweisen Gesundheitsbezogene Lebenskompetenz	Gesundheitsförderlich gestaltete schulische Rahmenbedingungen, Prozesse und Strukturen Gesundheitskompetente Schule	Bildungsförderlich gestaltete schulische Rahmenbedingungen, Prozesse und Strukturen

### Der verhaltensbasierte Ansatz.

Ab den 1970er- und 1980er-Jahren entwickelten sich vor allem kognitiv-verhaltensbasierte Interventionen, die den verhaltensbasierten Ansatz charakterisieren. Solche Maßnahmen sind in Schulen weitverbreitet. Sie sind oft standardisiert sowie von geringer Komplexität, bedienen ein auf kurzfristiges Handeln ausgerichtetes Interesse, fokussieren sich auf einzelne Themen und beziehen Betroffene im Sinne von Partizipation nicht mit ein. Maßnahmen, die heute mehr Anwendung finden, sind hingegen komplexerer Natur, denn sie sind auf strukturierte relativ stabile Muster der Lebensführung ausgerichtet, mittels derer sich Individuen mit den Anforderungen ihrer soziokulturellen Umwelt auseinandersetzen. Überwiegend verfolgen solche Maßnahmen heute einen ressourcenbezogenen partizipativen, am Empowerment^2^ orientierten Ansatz der Lebenskompetenzförderung (Life Skills) mit Elementen der Selbststeuerung des Gesundheitsverhaltens auf der Grundlage gesundheitswissenschaftlich fundierter Gesundheitsmodelle. „Klasse2000“ [22], „Erwachsen werden“ [23], „Be smart, don’t start“ [24] sind große, bundesweit verbreitete, z. T. extensiv evaluierte themenzentrierte Programme, die dort ihren Ausgang genommen und sich weiterentwickelt haben. Diese Maßnahmen haben allerdings, soweit erkennbar, in ihrer Planung und Umsetzung bislang wenig Berührungspunkte mit gesundheitsdidaktischen und -methodischen Entwicklungen und Positionsbestimmungen der Gesundheitspädagogik gehabt. Diese werden aktuell im schulpädagogischen Kontext im Ansatz der „Gesundheitskompetenz“ bzw. der „Gesundheitskompetenten Schule“ verstärkt diskutiert [25–27].

### Der Ansatz „Gesundheitsfördernde Schule“.

Initiierend und z. T. auch flankierend haben ab 1993 bis 2000 in Deutschland 2 große universell gesundheitsfördernde Schulmodellversuche auf ganz andere Weise Impulse zu tiefgreifenderen Veränderungen in der schulischen Gesundheitserziehung und Bildung gesetzt und zur Popularisierung des Konzeptes der schulischen Gesundheitsförderung beigetragen, indem sie die Schule als Setting in den Fokus der Gesundheitsförderung rückten („Netzwerk Gesundheitsfördernde Schulen“, 1993–1997 [28]; „Offenes Partizipationsnetz und Schulgesundheit – Gesundheitsförderung durch vernetztes Lernen“, „OPUS“, 1998–2000 [29, 30]). Von diesen aus hat sich dann ab 2003 bis 2008 mit über 40 nationalen Partnern im Modellprojekt „Anschub.de – Nationale Allianz für nachhaltige Schulgesundheit und Bildung“ [7] das Konzept „Gute Gesunde Schule“ entwickelt. Kernelement der „Gesundheitsfördernden Schule“ ist der Settingansatz, der in seiner Umsetzung geleitet wird durch die Prinzipien der Partizipation, des Empowerments und der Vernetzung, durch ein ganzheitliches Gesundheitsverständnis sowie durch eine ressourcenorientierte, salutogenetische Ausrichtung seiner verhältnisbezogenen Maßnahmen, die alle Mitglieder einer Schulgemeinschaft zu einem verantwortungsbewussten Umgang mit der eigenen Gesundheit und der ihrer Mitmenschen befähigen soll [1].

### Der Ansatz „Gute Gesunde Schule“.

Der Ansatz der „Guten Gesunden Schule“ geht über das Konzept der „Gesundheitsfördernden Schule“ hinaus und ist der anspruchsvollste und aktuellste in der Entwicklung schulischer Gesundheitsförderung. Er richtet seine Maßnahmen gezielt auf die Bildungsaufträge der Schule aus und unterstützt Schulen in diesem, heute so wichtig gewordenen Kernanliegen, qualitätsvolle pädagogische Arbeit zu leisten. Es sind die Verhältnisse, die mit Gesundheitsinterventionen bildungsförderlich zu gestalten sind. Damit kehrt sich die Blickrichtung der üblichen schulischen Gesundheitsförderung um: Es geht jetzt um die Bildungsförderung durch Gesundheit [3]. „MindMatters – mit psychischer Gesundheit gute Schule entwickeln“ ist ein Beispiel für ein Programm, das sich auf diesem Weg befindet [31].

Neben diesen Entwicklungssträngen hat sich ab Mitte der 2010er-Jahre das Konzept der „Health Literacy“ etabliert. Als „Gesundheitskompetenz“ hat es Eingang in die deutschsprachige Diskussion zur Praxis der schulischen Gesundheitsförderung gefunden und eine deutliche Resonanz erfahren, wenngleich seine praktische Umsetzung noch auf sich warten lässt [32].

## Schulische Gesundheitsförderung als Forschungsfeld

Eng damit verbunden, dass die schulische Gesundheitsförderung durch ihre Projektorientierung auf Umsetzung ausgerichtet ist und vorab intendierte Effekte nachzuweisen sind, erscheint die Evaluation als Forschungsfeld naheliegend. Aber hier gilt, was auch für die Gesundheitsförderung insgesamt gilt: Die Möglichkeiten der Evaluation von Maßnahmen werden oft nicht ausgeschöpft [20]. Zwar werden viele der Maßnahmen evaluiert, häufig sind sie aber unterfinanziert und beruhen auf einfachen Studiendesigns und Evaluationsansätzen, die Wirkungszusammenhänge dann nicht differenziert offenlegen können. Es fehlen häufig Vergleichsgruppen von Schulen, die ein RCT-Design^3^ vorsehen würde. In den vielfach projektorientierten Feldstudien lassen sich in der Regel aber keine zufälligen Zuordnungen von Schulen in Gesundheitsförderungs- und Vergleichsgruppen vornehmen, auch können Ergebnisverzerrungen nicht ausgeschlossen werden, da Störvariablen zumeist nicht ausreichend kontrolliert werden können oder wenn die Stichprobengrößen zu klein sind, was bei Settingprojekten mit Schulen oft der Fall sein wird [33].

Für Maßnahmen, die schulische Programme evaluieren und verhaltensbezogene Gesundheitsoutcomes mehrdimensional erfassen, gibt es inzwischen belastbare Ergebnisse internationaler Studien, die durch Reviews oder Metaanalysen abgesichert sind. Für den deutschsprachigen Raum fehlen solche Systematisierungen weitgehend [34, 35]. Es gibt aber Informationen zu wirksamen einzelnen Maßnahmen. Sie finden sich z. B. in der „Grüne Liste Prävention“ [36] oder in „die initiative – Gesundheit – Bildung – Entwicklung“. Dies ist eine niedersächsische Landesinitiative zur Verbreitung von qualitätsvollen Programmen und Maßnahmen zur Gesundheitsförderung in Schulen und Kindertageseinrichtungen (www.dieinitiative.de).

Was aus den Ergebnissen der Evaluationen für die Gestaltung zukünftiger Projekte oder für die Integration solcher Erkenntnisse in den Regelbetrieb der Schulen gelernt werden kann, ist ein weiteres Forschungsfeld [37]. Es geht dann um den Transfer, die Dissemination und Implementation solcher Erfahrungen, aber auch um die Frage, wie sinnvollerweise solche bei Settingprojekten komplexen Prozesse im Rahmen einer Interventionsplanung („logische Modelle“) zu konzipieren sind. Hier sind in den Jahren große Fortschritte erzielt worden, die gleichermaßen der Projekt- wie der Evaluationsplanung zugutekommen (z. B. Deming-Zirkel, Precede-Procede-Modell, Intervention-Mapping, s. [38]). Die Praxis bleibt hier hinter den Möglichkeiten zurück.

## Schulische Gesundheitsförderung als Politikfeld

Der schulischen Gesundheitsförderung sind durch die Präventionsgesetzgebung Ende der 1980er-Jahre und durch die fast gleichzeitige WHO-Initiative der „Gesundheitsfördernden Schulen“ neue Entfaltungsmöglichkeiten geschaffen worden.

Das Gesundheitsreformgesetz von 1989 hat die Grundlage für kassenseitige Förderungen mit dem § 20 Fünftes Buch Sozialgesetzbuch (SGB V) geschaffen, mit dem den gesetzlichen Kassen eine Zuständigkeit für Gesundheitsförderung zugeschrieben wurde, allerdings 1996 mit dem Beitragsentlastungsgesetz wieder zurückgenommen bzw. massiv eingeschränkt, mit gravierenden Folgen für die projektförmige Arbeit in den Schulen. Erst seit 2000 sind die Möglichkeiten wieder erweitert worden, zuletzt nach mehreren vergeblichen Anläufen, 2015 mit dem Gesetz zur Stärkung der Gesundheitsförderung und der Prävention [39, 40]. Bedeutsam ist auch das Fachkonzept „Mit Gesundheit gute Schulen entwickeln“ der Deutschen Gesetzlichen Unfallversicherung (2013; [41]), des neben den Kassen wichtigen Förderers der schulischen Gesundheitsförderung. Auf der Grundlage des § 14 Siebtes Buch Sozialgesetzbuch (SGB VII) folgt es dem Ansatz der integrierten Gesundheits- und Qualitätsentwicklung, bildet den Rahmen für die Arbeit des Sachgebietes „Schulen“ und hält damit Anschluss an die aktuellen konzeptionellen Entwicklungen. Walter et al. [42] geben zu diesen Entwicklungen und Umsetzungen einen umfassenden Überblick, einschließlich der Gesetze des Öffentlichen Gesundheitsdienstes wie auch für die Umsetzungen in der Bildungspolitik auf Länderebene für die Schulen.

Für die schulische Prävention und Gesundheitsförderung ist in den letzten Jahren der 2012 veröffentlichte Beschluss der Kultusministerkonferenz (KMK) „Empfehlung zur Gesundheitsförderung und Prävention in der Schule“ richtungsweisend geworden, weil er unmittelbar aus bildungs- und schulpolitischer Perspektive die letzten konzeptionellen Entwicklungen aufgreift und feststellt: „Gesundheitsförderung und Prävention sind integrale Bestandteile von Schulentwicklung. Sie stellen keine Zusatzaufgaben der Schulen dar, sondern gehören zum Kern eines jeden Schulentwicklungsprozesses“ [43]. Diese Empfehlung schließt auch an die KMK-Empfehlung von 1992 „Zur Situation der Gesundheitserziehung in der Schule“ an. Dort heißt es noch: „Gesundheitserziehung gilt in den Ländern als wesentlicher Bestandteil des Bildungs- und Erziehungsauftrages der Schule“ [44]. Erstmals wird dort auch die Verknüpfung der verschiedenen Themen und Bereiche der schulischen Gesundheitserziehung in einem Konzept der „Gesunden Schule“ vorgestellt. Die Brücke zum Settingansatz war damit hergestellt, ist aber erst 20 Jahre später vertieft und erweitert wieder aufgegriffen worden.

Nicht unerheblich sind in diesem Zusammenhang die wenig beachteten Beschlüsse der 336. Sitzung des Schulausschusses der KMK vom 09.–10.03.2000. Denn hier sind wegweisende Entscheidungen gefallen. Es wurde festgestellt, dass der Schulausschuss gegenwärtig keinen Bedarf an einer Überarbeitung des Berichts der Kultusministerkonferenz vom 05.–06.11.1992 sieht, obwohl inzwischen 2 große bundesweite settingbasierte Schulmodellversuche, gefördert von der Bund-Länder-Kommission für Bildungsplanung und Forschungsförderung (BLK), erfolgreich gelaufen waren (s. oben). Es wurde beschlossen, dass die KMK sich an das Bundesministerium für Gesundheit (BMG) mit der Bitte um einen Erfahrungsaustausch zwischen Bund und Ländern zur Gesundheitserziehung wenden möge und dass dabei die Bundeszentrale für gesundheitliche Aufklärung (BZgA) die Funktion einer zentralen Informationsvermittlung übernimmt. Daraus ist der Erfahrungsaustauch der Länderreferent*innen in den Kultusministerien und Senatsbehörden entstanden, der seitdem mehrfach im Jahr, koordiniert und moderiert von der BZgA, zusammenkommt. Schließlich wird ein Beschluss zur Frage der Einrichtung eines eigenständigen Faches „Gesundheitserziehung“ gefasst, der bis heute Gültigkeit hat. Dort heißt es: „Er [der Schulausschuss] schließt sich dem Votum der Fachreferent*innen der Länder für Fragen der Gesundheitserziehung an, dass die Einrichtung eines eigenständigen Faches „Gesundheitserziehung“ abzulehnen sei“ (S. 10).

Der Stand und die Entwicklung des Themas Gesundheit in den Schulen lassen sich an den Schulgesetzen der Länder ablesen, insbesondere aber an den Konzeptionen von „guten Schulen“, die die Kultusministerien und obersten Senatsbehörden der Länder in ihren jeweiligen Referenzrahmen zur Schulqualität dokumentiert haben. Denn dort wird deutlicher als in den Schulgesetzen, wie und in welchem Umfang das Thema Gesundheit mit der Qualität und Qualitätsentwicklung der Schulen in den Ländern verknüpft wird. Eine systematische Analyse hierzu haben Paulus und Petzel (2021; [45]) vorgelegt.

Ohne auf die Details dieser Analyse der Referenzrahmen Schulqualität der Länder zum Thema Gesundheit einzugehen, können folgende Ergebnisse festgehalten werden:

- Das Gesundheitsverständnis, auf das sich der jeweilige Referenzrahmen der Länder bezieht, wird in den Referenzrahmen selten expliziert.
- Als wesentlicher Akteur wird häufig grob „die Schule“ benannt.
- In der Hälfte der Referenzrahmen wird auch die Schulleitung als Akteur benannt und damit die zentrale Rolle der Schulleitung als „Herbeiführer des Wandels“ (Change Agent) betont.
- Mehrmals nehmen die Referenzrahmen auf das mehrdimensionale, theoretisch und empirisch abgesicherte Modell der Schulqualität von Ditton (2002; [46]) Bezug.
- Auf Schulebene werden – ohne spezifische Adressat*innen zu benennen – Prävention und Gesundheitsförderung in knapp der Hälfte der Bundesländer als Maßnahmen explizit erwähnt.
- Verhaltens- und Verhältnisorientierungen werden auf der Ebene der Referenzrahmen nur in geringem Umfang erwähnt.
- Die Gesundheit der Schüler*innen wird in ihrer subjektiven bzw. objektiven Ausprägung, in den Dimensionen von Gesundheit, in den Maßnahmen mit Themen und Handlungsfeldern in den Referenzrahmen in der Hälfte der Länder repräsentiert. Es zeichnet sich aber kein erkennbarer Trend oder ein systematisches Profil ab.
- Gleiches gilt auch für die Widerspiegelung der gesundheitlichen Situation der Lehrkräfte. Auch hier ist keine Systematik im Vergleich der Länder erkennbar, mit Ausnahme der Nennungen zum „Sicherheits‑, Arbeits- und Gesundheitsschutz; Gefährdungsbeurteilung“. Hier können 9 von 16 Ländern mit einer entsprechenden Nennung registriert werden.
- Schulleitungen als Betroffene (statt als Akteure) kommen in den Referenzrahmen in sehr geringem Maße vor.
- Gleiches gilt für das sonstige schulische Personal, worunter dann in der Regel weiteres pädagogisch tätiges Personal verstanden wird (z. B. Schulpsycholog*innen, Schulsozialarbeiter*innen).

Deutlich wird in dieser Auflistung, dass sich auf der Ebene der Vorstellungen der Bundesländer zur Qualität schulischer Gesundheitsförderung (noch) kein einheitliches Bild ihrer Ausgestaltung und Umsetzung erkennen lässt. Jenseits dieser allgemeinen Zusammenfassung werden bei vertiefenden Analysen der Qualitätskonzepte der Länder Verknüpfungen mit der pädagogischen Qualitätsentwicklung und dem Qualitätsmanagement der Schulen erkennbar (z. B. Thüringen, Rheinland-Pfalz, Hessen), die Anknüpfungspunkte für Weitentwicklungen bieten.

## Chancen und Herausforderungen schulischer Gesundheitsförderung

Nachfolgend werden wichtige Punkte zusammengestellt, die auf der Grundlage des bisher Erreichten für die Weiterentwicklung der schulischen Gesundheitsförderung von Bedeutung sind.

- Eine systematische Bestandsaufnahme und -analyse der präventiven und gesundheitsfördernden Maßnahmen im Kontext Schule ist nötig, um die Qualitätsentwicklung der schulischen Gesundheitsförderung absichern zu können. Es existieren zwar bereits verschiedene Angebotsdatenbanken^4^ oder der jährlich erscheinende Präventionsbericht der GKV (Spitzenverband Bund der Krankenkassen) (2021; [39]), der Daten liefert, aber das reicht längst nicht aus.
- Bei der Dissemination und Implementation von Projektergebnissen oder Maßnahmen ist vorab sicherzustellen, dass „Modelle guter Praxis“ auch in eine „Praxis der guten Modelle“ überführt werden. Viele gute Projekte kommen über ihr Ende nicht hinaus, werden keine Programme, die Eingang in den Regelbetrieb finden. Es entsteht eine ermüdende „Projektitis“.
- Für eine nachhaltige qualitätsgesicherte Wirksamkeit der schulischen Gesundheitsförderung sind deshalb auch multimodale und multithematische Maßnahmen zu bevorzugen, die durch ein strukturiertes schulisches Gesundheitsmanagement aufeinander bezogen und in das Bildungsmanagement der Schule integriert werden können [45]. Schulen sind in solchen Vorhaben zu unterstützen.
- Solche das ganze Setting Schule betreffenden Interventionen müssen angesichts der großen Vielfalt der Schulformen und -arten (u. a. Förderschulen, berufsbildende Schulen, Ganztagsschulen) stärker als bisher auf die jeweilige Schule sowie ihre Lehrer- und Schüler*innenschaft individuell zugeschnitten sein („taylored interventions“) bzw. ihr ermöglichen, einen spezifischen Zuschnitt zu entwickeln [47].
- Der Ansatz der Förderung der „Gesundheitskompetenz“ ist darauf hin zu prüfen, inwieweit sein Verständnis von Kompetenz kompatibel ist mit dem, welches im Rahmen einer kompetenzorientierten Pädagogik der Schule [48] diskutiert wird, um Missverständnisse und Fehlentwicklungen zu vermeiden. Dies gilt auch für die Klärung der Ähnlichkeit zum Ansatz der „Gesundheitsfördernden Schule“ oder auch der „Guten Gesunden Schule“, wenn das Verständnis der Gesundheitskompetenz auch auf eine organisationale „Gesundheitskompetente Schule“^5^ erweitert wird. Zu klären ist dann ggf. auch, ob sich die Förderung der Gesundheitskompetenz als integraler Bestandteil schulischer Bildungsarbeit verstehen lässt, der die Forderung nach der Einrichtung eines Schulfaches Gesundheit und die Neufassung einer zeitgemäßen Gesundheitspädagogik und -didaktik begründen kann.
- Auch die Umsetzung der in 2015 verabschiedeten „Sustainable Development Goals“ (dt.: Ziele für nachhaltige Entwicklung) im schulischen Kontext ist von großer Relevanz für die schulische Gesundheitsförderung, insbesondere für eine zeitgemäße Gesundheitsbildung [25]. Einige der 17 Ziele sprechen explizit Themen an, die hier relevant sind, z. B. Ziel 1: keine Armut, Ziel 4: gute Gesundheit; Ziel 5: qualitätsvolle Bildung; Ziel 11: nachhaltige Städte und Kommunen.
- In Verbindung mit Artikel 25 „Gesundheit“ der 2007 verabschiedeten Behindertenrechtskonvention der Vereinten Nationen ist nach wie vor darauf hinzuarbeiten, dass die Inklusion im Schulsystem umgesetzt wird und die Entfaltung der Gesundheitspotenziale aller Schüler*innen im Rahmen einer inklusiv gestalteten Pädagogik gewährleistet ist [49].
- Standards und Indikatoren, wie sie auf europäischer und weltweiter Ebene von der WHO z. Zt. systematisiert werden, könnten, in Deutschland eingeführt und angepasst, einen wichtigen Beitrag zur strukturierten Qualitätsentwicklung der schulischen Gesundheitsförderung leisten [50].
- Durch die vorherrschende Projektorientierung fehlt es allerdings an übergreifenden Konzeptionen, die die verschiedenen Themen und Trends aus dem Schulbildungs- und Gesundheitsbereich verbinden und diese Schnittstelle vertiefend theoriebasiert schließen. Es fehlen auch solche, die sich der Kluft widmen, die sich zwischen einer evidenzbasierten Praxis für die Schule und einer praxisbasierten Evidenz in der Schule auftut.
- Eine stärkere Verknüpfung der schulischen Gesundheitsförderung mit Entwicklungen in der Schulpädagogik, insbesondere mit der sich etablierenden Gesundheitsbildung in Absetzung von der traditionellen Gesundheitserziehung, könnte eine solcher Verbindungen darstellen, weil so die Verknüpfung mit Ansätzen der pädagogischen Schulentwicklung geleistet werden kann [25, 26].
- Die schulische Gesundheitsförderung braucht darüber hinaus eine Plattform für den Austausch über Konzept‑, Theorie- und Empirieentwicklung, die auch politische Positionierungen ermöglichen kann. Ein jährlich stattfindender Kongress, an dem sich die Akteure aus Praxis, Forschung, Politik, aus den Medien und der Zivilgesellschaft einfinden, könnte ein solcher Ort sein. Der Austausch könnte in einem jeweils fortzuschreibenden „Masterplan“ zur Orientierung für die weitere Entwicklung der schulischen Gesundheitsförderung münden.
- Für die schulische Prävention und Gesundheitsförderung ist der Auftrag, sozial bedingte gesundheitliche Ungleichheiten, die z. T. mit einer Migrationsproblematik verknüpft sind, zu verringern, immer noch eine Herausforderung. Hauptschulen, Förderschulen und berufsbildende Schulen, die zumeist von einer erhöhten Anzahl sozial benachteiligter Schüler*innen besucht werden, sind deutlich in den Maßnahmen unterrepräsentiert. Hier bleibt dann immer aber noch der Hinweis wichtig, dass dieser Auftrag auch eine gesamtgesellschaftliche Aufgabe ist. Nur durch gemeinsame und aufeinander abgestimmte Anstrengungen und unter Einbeziehung der auf den verschiedenen gesellschaftlichen Ebenen relevanten Akteure, einschließlich der Schüler*innen selbst, erscheint ein solcher Ausgleich unter Mitwirkung der Schule möglich.

## Fazit

Vor dem Hintergrund der hier skizzierten 30-jährigen Entwicklung und ihrer einzelnen Chancen und Herausforderungen wird eines sichtbar: Es fehlt eine Rahmung für die schulische Gesundheitsförderung, die theoriegeleitet Forschung und Praxis miteinander anleitet und verbindet. Ein Verständnis von schulbezogenem Gesundheitsmanagement, das integriert und mit dem pädagogischen Qualitätsmanagement und der pädagogischen Qualitätsentwicklung der Schule verbunden ist, könnte eine vereinheitlichende Perspektive bilden, die Ressourcen bündelt und gezielt den Fortschritt für die Einzelschule, aber auch für Netzwerke von Schulen ermöglicht, die sich als von- und miteinander lernende Organisationen dem Ziel einer guten Schule nähern.

## Footnotes

1 https://www.destatis.de/DE/Service/Bibliothek/fachserienliste-artikel.html; (Fachserie 11, Reihe 1 und 2); Zugriffsdatum: 25.04.2022.

2 Empowerment: Strategien und Maßnahmen zur Stärkung von Autonomie und Selbstbestimmung des Menschen.

3 RCT: randomisierte kontrollierte Studie.

4 Angebotsdatenbanken sind beispielsweise die Praxisdatenbank des Kooperationsverbundes Gesundheitliche Chancengleichheit (www.gesundheitliche-chancengleichheit.de/praxisdatenbank/) und die Datenbank von Deutschlands Initiative für gesunde Ernährung und mehr Bewegung (www.in-form.de).

5 Vgl. Modellvorhaben „Gesundheitskompetente Schule: Organisationsentwicklung für die Stärkung der Gesundheitskompetenz im Setting Schule“ (GeKoOrg-Schule, https://gekoorg-schule.de/).
